# Treatment With Naringenin Elevates the Activity of Transcription Factor Nrf2 to Protect Pancreatic β-Cells From Streptozotocin-Induced Diabetes *in vitro* and *in vivo*

**DOI:** 10.3389/fphar.2018.01562

**Published:** 2019-01-28

**Authors:** Rashmi Rajappa, Dornadula Sireesh, Magesh B. Salai, Kunka M. Ramkumar, Suryanarayanan Sarvajayakesavulu, SubbaRao V. Madhunapantula

**Affiliations:** ^1^Department of Water & Health, Faculty of Life Sciences, JSS Academy of Higher Education and Research, Mysuru, India; ^2^SRM Institute of Science and Technology, Chennai, India; ^3^Center of Excellence in Molecular Biology & Regenerative Medicine, Department of Biochemistry, JSS Medical College, JSS Academy of Higher Education and Research, Mysuru, India

**Keywords:** diabetes, streptozotocin, naringenin, Nrf2, MIN6 cells, apoptosis

## Abstract

Chronic hyperglycemia and unusually high oxidative stress are the key contributors for diabetes in humans. Since nuclear factor E2-related factor 2 (Nrf2) controls the expression of antioxidant- and detoxification genes, it is hypothesized that targeted activation of Nrf2 using phytochemicals is likely to protect pancreatic β-cells, from oxidative damage, thereby mitigates the complications of diabetes. Naringenin is one such activator of Nrf2. However, it is currently not known whether the protective effect of naringenin against streptozotocin (STZ) induced damage is mediated by Nrf2 activation. Hence, the potential of naringenin to activate Nrf2 and protect pancreatic β-cells from STZ-induced damage in MIN6 cells is studied. In MIN6 cells, naringenin could activate Nrf2 and its target genes GST and NQO1, thereby inhibit cellular apoptosis. In animals, administration of 50 mg/kg body weight naringenin, for 45 days, significantly decreased STZ-induced blood glucose levels, normalized the lipid profile, and augmented the levels of antioxidants in pancreatic tissues. Immunohistochemical analysis measuring the number of insulin-positive cells in pancreas showed restoration of insulin expression similar to control animals. Furthermore, naringenin promoted glycolysis while inhibiting gluconeogenesis. In conclusion, naringenin could be a good anti-diabetic agent, which works by promoting Nrf2 levels and by decreasing cellular oxidative stress.

## Introduction

Diabetes is a non-communicable disease with multiple etiological factors resulting from a defect in insulin secretion, insulin action, or both, leads to chronic hyperglycemia with disturbances in the metabolism of carbohydrates, lipids, and proteins (American Diabetes and Association, 2010). Despite various cost-effective treatment strategies and public campaigns highlighting the key risk factors for diabetes, the incidence and burden are increasing at alarming rates with an estimated 422 million individuals presently suffering from this disease globally. According to the IDF, the number of diabetics is predicted to increase to 642 million by 2040 (American Diabetes and Association, 2015). A decrease in the number of insulin-producing functional β-cells and alterations in β-cell mass contributes to the pathophysiology of both type1 and type 2 diabetes (Cerf, 2013). Recent studies have identified that oxidative stress, caused by excess ROSs, is one of the most important causing factors for diabetes complications (Forbes and Cooper, 2013). Moreover, since pancreatic β-cells express very low antioxidant defense enzymes, they are more susceptible to oxidative stress caused by (a) free radicals; (b) misfolded proteins; and (c) endoplasmic reticulum hyperactivity (Lenzen et al., 1996). As a result of this cellular stress, β-cells undergo apoptosis, culminating in pancreatic dysfunction (Kajimoto and Kaneto, 2004). Therefore, promoting the expression of genes coding for antioxidant enzymes appears to be a possible therapeutic approach against stress-associated cell damage in pancreatic β-cell (Qin and Hou, 2016).

Nuclear factor erythroid 2-related factor-2 (Nrf2) is a key leucine zipper transcription factor that regulates the expression of intracellular antioxidant enzymes thereby prevent the loss of cells due to oxidative stress (Lacher et al., 2015). Under basal conditions, Nrf2 exists in its inactive state due to its association with Keap1 (Kelch-like erythroid-cell-derived protein with CNC homology [ECH]-associated protein) (Ma and He, 2012). Mechanistically, Keap1 anchors Nrf2 in the cytoplasm and target it for proteasomal degradation by promoting its association with the Cullin-3 (Cul3)/Ring box-1 (Rbx1) E3 ligase system (Ma and He, 2012). However, when cells are exposed to oxidative or electrophilic stress, the reactive cysteines of Keap1 undergo modification, causing dissociation of the Nrf2 from Keap1 complex, allowing its’ translocation into the nucleus. Nuclear Nrf2 binds to antioxidant response elements (ARE) sequences and trigger the expression of genes involved in combating cellular stress (Jung and Kwak, 2010). These stress-response genes include NADPH quinone oxidoreductase (NQO1), Heme oxygenase-1 (HO-1), glutathione S-transferase (GST), superoxide dismutase (SOD), catalase (CAT), and γ-glutamylcysteine synthetase (GCS) (Jung and Kwak, 2010). Since Nrf2-dependent cellular defense response can protect organs, activation of Nrf2 using phytochemical has been implicated as a strategy to combat diseases such as diabetes (Lu et al., 2016).

**FIGURE 1:**
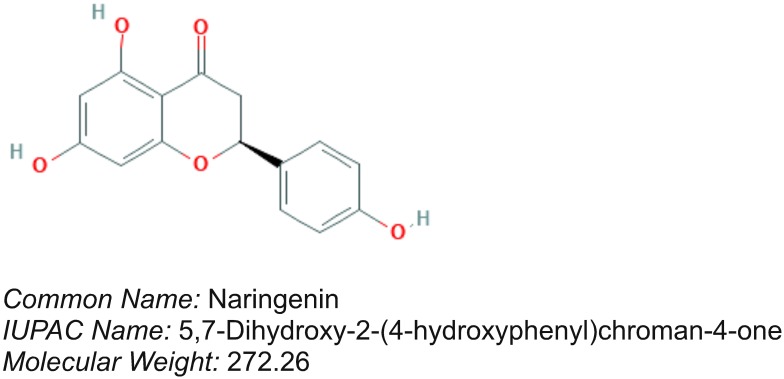
Chemical structure of naringenin. Naringenin is a flavonoid present in citrus species fruits.

Naringenin (Figure 1) is a bioactive flavonoid predominantly present in citrus fruits such as grapes, blood orange, lemons, and tomatoes (Sumathi et al., 2015). Prior studies have demonstrated that naringenin treatment could offer protection against (a) ethanol-induced hepatotoxicity and (b) cisplatin-induced nephrotoxicity (Sumathi et al., 2015). For instance, naringenin has been shown to down-regulate the phosphorylation of MAPK and nuclear factor kappaB (NFκB) subunit p65 in daunorubicin-induced nephrotoxicity by preventing the epidermal growth factor receptor (EGFR)-phosphoinositide-3 kinase (PI3K)-Akt/extracellular signal regulated kinase (ERK) MAPK signaling (Karuppagounder et al., 2015). Recently, naringenin has been shown to activate Nrf2 (Esmaeili and Alilou, 2014). Esmaeili and Alilou (2014) reported that naringenin suppressed the hepatic inflammation by stimulating the Nrf2 pathway in carbon tetrachloride treated rats. In addition, naringenin provided protection against 6-hydroxy dopamine-induced oxidative stress in SH-SY5Y cells (Lou et al., 2014). However, not much is known about whether naringenin reduces oxidative stress in MIN6 pancreatic β-cells through Nrf2 signaling. Therefore, in the current study, we have determined the Nrf2 activation potential of naringenin in MIN6 cells *in vitro*; and assessed its protective effect against streptozotocin (STZ)-induced pancreatic β-cell apoptosis. Furthermore, the anti-diabetic and anti-oxidative effects of naringenin were studied using male Wistar albino mice treated with multiple low doses of STZ. In conclusion, data from our studies report the ability of naringenin to activate Nrf2 thereby provide protection against STZ-induced cell death *in vitro* as well as in experimental animal models.

## Materials and Methods

### Culturing of MIN6 Cells

MIN6 is a mouse insulinoma cell line obtained from National Centre for Cell Science (NCCS), Pune, India. MIN6 display many important characteristics that are similar to pancreatic islets (Ishihara et al., 1993). For example, MIN6 cells exhibit glucose-stimulated insulin secretion (GSIS) (Cheng et al., 2012). MIN6 cells were cultured in DMEM supplemented with 10% heat-inactivated FBS, 100 U/mL penicillin, 100 μg/mL streptomycin, and 2.0 mM glutamine (Purchased from GE Healthcare, Little Chalfont, United Kingdom) in a carbon dioxide incubator maintained at 37°C with 5% CO_2_. MIN6 cells with passage number between 5 and 20 were used for all the experiments (Elango et al., 2016).

### Determination of Cell Viability Using MTT Assay

The effect of naringenin on the viability of MIN6 cells was measured using an MTT assay (Mosmann, 1983). Experimentally, first, MIN6 cells (2 × 10^4^ cells/well) were plated in 96-well plates and allowed to grow for 24 h in a CO_2_ incubator. Next, the growing cells were exposed to increasing concentrations (0–200 μM) of naringenin (Sigma Chemical Company, St. Louis, MO, United States) for 24 h at 37°C. After treatment, cells were replenished with 90 μL phenol-red free media containing 10 μL MTT (5 mM) and incubated for additional 3 h in the CO_2_ incubator. Media was aspirated, the precipitate was dissolved in 50 μL DMSO, and the absorbance measured at 540 nm using a plate reader (Infinite 1000, Tecan, Mannedorf, Switzerland). The experiments were performed in triplicate. The relative cell viability (%) compared to control cells treated with DMSO was calculated using: Cell viability (%) = (A_sample_-A _blank_)/A_control_-A_blank_) × 100. Since an about 35% cell death was observed at 200 μM, subsequent studies were conducted with naringenin concentrations < 200 μM.

To study the protective role of naringenin on STZ-induced cytotoxicity, first, the MIN6 cells were pretreated with increasing concentration of naringenin (0–100 μM) for 24 h. Next, the naringenin-treated cells were exposed to 10 mM STZ (Primary stock of 1.0M was prepared by dissolving in 0.1M Citrate buffer pH 4.5 followed by the addition of DMSO) for 1 h and the number of viable cells estimated using MTT assay. All experiments were performed in triplicates.

### Evaluation of the Potential of Naringenin to Activate Nrf2 Using Nrf2-Keap1 Complementation System

2 × 10^4^ MIN6 cells/ml were transiently transfected with Nrf2-Keap1 complementation system in a 12-well plate using Lipofectamine-2000 according to the manufacturer’s protocol (Invitrogen, Carlsbad, CA, United States). Six hours after transfection, the media was replaced with a fresh batch of medium, and cells treated with naringenin (25, 50, 100 μM) for 24 h. Control and treated cells were lysed in 1X lysis buffer (pH 7.8; Promega, Madison, WI, United States), protein lysates collected, and the debris separated by centrifugation at 10,000 *g* at 4°C for 5 min. Total protein was estimated using the Bradford reagent (Bio-Rad Laboratories Inc, Hercules, CA, United States). Next, 100 μL luciferase substrate (prepared by mixing 10 ml of luciferase assay buffer with the lyophilized Luciferin; Promega, Madison, WI, United States) was added to the 20 μL of supernatant containing 175.0 μg of total protein and the luciferase activity measured using a luminometer (Promega, Madison, WI, United States). The developed sensor system detects the potential of naringenin to stimulate the Nrf2-Keap1 complex dissociation. A fall in luciferase signal is inversely proportional to the activation of Nrf2. The results were presented as fold change of three independent experiments.

### Separation of Nuclear and Cytosolic Fractions Using Pierce NE-PER Kit

To check the effect of naringenin on Nrf2 translocation, nuclear and cytoplasmic extracts were separated using Pierce NE-PER^®^ kit according to the manufacturer’s guidelines (Pierce, Rockford, IL, United States). In brief, cells (2 × 10^4^ MIN6 cells/ml) were homogenized in CER-I buffer vigorous vortexing in the pre-extraction buffer and incubated on ice for 15 min. The cellular homogenate was centrifuged at 10,000 × *g* for 10 min at 4°C and the supernatant containing the cytoplasmic fraction separated. Next, nuclei present in the pellet were suspended in NER buffer (provided with protease inhibitors) and the nuclear fraction generated by thorough vortex mixing for 1 min with a break for every 10 min for 40 min. The vortexed samples were centrifuged at 16,000 × *g* for 15 min at 4°C, to separate the supernatant containing a nuclear fraction. The separated nuclear fraction was used for western blotting.

### Estimation of Total Protein and Western Blotting

Total protein content in the cytoplasmic and nuclear fractions was quantified with the Bradford method using BSA as a standard (Kruger, 2009). Samples (100 μg) were denatured using sample buffer at 95°C for 5 min, proteins were separated on 4–12% SDS-PAGE gel (Bio-Rad, Philadelphia, PA, United States) and electroblotted onto a nitrocellulose membrane (Schleicher & Schuell, Keene, NH, United States). Primary and secondary antibodies against Nrf2, caspase-3, β-actin and lamin-B (Santa Cruz Biotechnology, Santa Cruz, CA, United States) were used to detect corresponding proteins. The blot was developed using ECL (Bio-Rad, Philadelphia, PA, United States) and the signals captured using a gel documentation system (GBOX, Syngene, United Kingdom).

### ARE-Luciferase Reporter Gene Assay

NQO1-ARE-Luc and GST-ARE-Luc reporter constructs, given by Donna D. Zhang (College of Pharmacy, University of Arizona, Tucson, AZ, United States) to Dr. Ramkumar, were used for cell-based reporter gene assay. For more details about the construction of these reporter plasmids, readers can refer to (Ramkumar et al., 2013). ARE-Luc constructs (500 ng/well) were transiently transfected into MIN6 cells in 12-well plates using Lipofectamine-2000 as described previously (Ramkumar et al., 2013). After 6 h of transfection, the media was changed and the cells exposed to 25, 50, and 100 μM naringenin for 24 h. The cell lysates were collected and the luminometric assay was carried out according to Ramkumar et al. (2013). The increase in the luciferase activity compared to control DMSO-treated cells was represented. The results were represented as the averages of at least three independent experiments.

### Measurement of Intracellular Reactive Oxygen Species

Levels of intracellular ROS were determined by flow cytometry using an oxidation-sensitive fluorescent dye, 2,7-dichlorodihydrofluorescein diacetate (H_2_DCFDA) (Eruslanov and Kusmartsev, 2010). Experimentally, first, MIN6 cells (2 × 10^4^ cells/well in 100 μL) were treated with 50 and 100 μM naringenin in complete medium for 24 h. Next, naringenin-treated cells were exposed to 10 mM STZ for 1 h and the cells were incubated with 20 μM H_2_DCFDA (10 μL) for 30 min at 37°C. The reaction was terminated by washing with phosphate-buffered saline (PBS) containing 10% fetal calf serum (FCS). Cells were centrifuged at x800 *g* for 10 min, washed, and resuspended in 1ml PBS. The fluorescence intensity was measured using FACS (BD Biosciences, San Jose, CA, United States). Data were analyzed using BD Cell QuestTM Pro Analysis software and the shift in fluorescence intensity caused by DCF production, which is an indicator of ROS generation, represented as a histogram.

### Annexin V-FITC/PI Double Staining and Analysis by Flow Cytometry

To assess the efficacy of naringenin for protecting MIN6 cells from STZ-induced cell death, the FITC Annexin V/Dead Cell Apoptosis Kit (Invitrogen), was used (Hingorani et al., 2011). Experimentally, MIN6 cells were cultured in 6-well plates and treated with naringenin (50 and 100 μM) for 24 h. Next, naringenin-treated cells were exposed to 10 mM STZ. After 1 h, the cells were trypsinized, collected and washed with cold PBS. The supernatant was discarded and the cells resuspended in 200 μL of the 1X binding buffer, containing 50 mM HEPES, 700 mM NaCl, 12.5 mM CaCl_2_, pH 7.4. To 100 μL cell suspension, 5 μL annexin-V-FITC and 1 μL of PI solution (100 μg/mL) were added. Then, the cells were incubated for 15 min at room temperature in dark, and 400 μL of 1X annexin-binding buffer added. The stained cells were examined by flow cytometry using Cell QuestTM Pro Analysis software.

### Determination of Protective Effects of Naringenin in Mice

Animal experiments were conducted after receiving approval from the JSS Medical College Institutional Animal Ethics Committee (JSSMC/IAEC/18/5675/DEC2013), Mysuru, India. In brief, male albino mice of 4–6 weeks old, weighing about 25–30 g, were procured from the Central Animal Facility, JSS Medical College and were maintained under standard laboratory conditions: viz., temperature (25 ± 2°C) and humidity with an alternating 12 h-12 h light/dark cycles.

#### Experimental Induction of Diabetes

Experimental diabetes was induced by multiple low dose streptozotocin (MLD-STZ) injections according to Elango et al. (2016). In brief, STZ was dissolved in 0.1M citrate buffer (pH-4.5) and injected 50 mg/kg/day intraperitoneally for 5 successive days. Blood (50 μL) was collected from the tail vein and used for estimating glucose using a glucometer (Accu-Check Active). Mice with blood glucose concentration > 250 mg/dl were selected for testing the potency of naringenin for mitigating STZ-induced cell damage. First, 24 STZ treated and 12 normal mice were divided into a total of 6 groups as shown in Table 1. Next, naringenin 50 mg/kg and 100 mg/kg body weight (prepared in 0.5% carboxymethyl cellulose) was administered orally to control and experimental groups for 45 days. At the end of the experiment (after 24 h of last naringenin administration), animals were deprived of food overnight and euthanized using chloroform and sacrificed by decapitation (Cenedella et al., 1975). Blood was collected by cardiac puncture in all experimental animals and allowed for clotting. Serum was separated by centrifuging at 3000 rpm for 15 min at room temperature and stored at −20°C until analysis. Glibenclimide (600 μg/kg body weight; prepared in 0.5% carboxymethyl cellulose) was used as a reference drug in this study.

**Table 1:** Control and experimental groups of mice.

Group	No. of mice	Category	Treatment agent	Dose and frequency of the treatment agent (mg/kg b.w.)	Route of administration	Comment
I	6	Control	Vehicle: Carboxy methyl cellulose (CMC)	0.5%, Every day for 45 days	Oral	Vehicle control
II	6		Naringenin^∗^ (NAR)	100 mg/kg, every day for 45 days	Oral	Naringenin control
III	6	STZ	Streptozotocin (STZ)	50 mg/kg, 5 consecutive days	Intra peritoneal	STZ control
IV	6		STZ, followed by NAR^∗^	STZ – 50 mg/kg, 5 consecutive days NAR – 50 mg/kg, every day for 45 days	Oral	STZ mice treated with low dose naringenin
V	6		STZ, followed by NAR^∗^	STZ – 50 mg/kg, 5 consecutive days NAR – 100 mg/kg, every day for 45 days	Oral	STZ mice treated with high dose naringenin
VI	6		STZ, followed by Glibenclamide^∗^	STZ – 50 mg/kg, 5 consecutive days GLC – 600 μg/kg, every day for 45 days	Oral	A positive control group

*^∗^Naringenin (NAR) and glibenclamide (GLC) were suspended in 0.5% CMC.*

#### Analytical Procedure

Fasting blood glucose levels were monitored using Accu-Chek Active Glucometer every 3rd day. IPGTT was carried out at the completion of the experimental period (Goren et al., 2004). Briefly, after 12 h fasting, the mice were intraperitoneally injected with glucose (1 g/kg b.w) and blood glucose measured at 30, 60, 90, and 120 min. Serum insulin level was determined using mouse ELISA kit (Crystal Chem Inc.).

The collected blood was used for the estimation of total cholesterol (TC), triglycerides (TG), high-density lipoprotein (HDL)-cholesterol using kits available from Coral Clinical System, Goa, India. Phospholipids and free fatty acids were determined according to the protocols described by Falholt et al. (1973) and Stewart (1980). Very low-density lipoprotein (VLDL)-cholesterol and low-density lipoprotein (LDL)-cholesterol were calculated by Friedwald formula. VLDL = TG/5; LDL = TC-(HDL + VLDL).

The level of lipid peroxidation, in terms of TBARS formed, and hydroperoxides content was measured using the methods described by Niehaus and Samuelsson (1968) and Jiang et al. (1992), respectively. Further, antioxidant enzymes (a) SOD by Kakkar et al. (1984); (b) CAT by Sinha (1972); (c) GPX by Rotruck et al. (1973); and GSH S-Transferase by Habig et al. (1974) and antioxidant molecules such as reduced GSH by Ellman (1959) were measured in the pancreatic tissues.

#### Estimation of Carbohydrate Metabolic Enzymes and Glycogen Content in Liver of Control and Experimental Mice

Hexokinase, Glucose-6-phosphatase (G6-P) and Fructose-1,6-bisphosphatase (F1,6-BP) were assayed according to the protocols described by Brandstrup et al. (1957); Koide and Oda (1959), and Gancedo and Gancedo (1971) respectively. The inorganic phosphate (Pi) liberated was estimated by the method of Fiske and Subbarow (1925). Glucose-6-phosphate dehydrogenase (G6-PD) was determined by the method of Ells and Kirkman (1961). Glycogen content was estimated by the method of Morales et al. (1973) and protein content in tissue homogenates was measured by the method of Lowry et al. (1951).

#### Histology of Pancreas

Pancreatic tissues were fixed in 10% buffered neutral formalin for 24 h and dehydrated using ethanol and xylene for 30 min each. Tissues were then incubated in liquid paraffin (58°C) twice for 60 min each. Tissue blocks were sectioned (5 μm thickness) and stained using hematoxylin and eosin (Feldman and Wolfe, 2014). The pancreatic sections were analyzed for morphological changes using a microscope (Carl Zeiss, Thornwood, NY, United States). The photomicrographs representing the overall assessment were captured and showed in the figures.

#### Immunohistochemical Staining for Insulin

To examine the expression of insulin in pancreatic tissues, an immunohistochemical examination was carried out with insulin antibody (SC-9168; Santa Cruz Biotechnology, Heidelberg, Germany). The 5 μm thick paraffin sections were deparaffinized in xylene and hydrated with ethanol. Then the hydrated sections were treated with 3% H_2_O_2_ in methanol for 30 min to block any endogenous peroxidase. The sections were washed at least three times with 0.01M phosphate buffer (pH 7.4) for 10 min; and processed further by an indirect immunoperoxidase technique using a One-Step Polymer-HRP Detection kit (Leica Biosystems, Newcastle, United Kingdom) with secondary antibodies. The sections were counterstained with hematoxylin. The stained sections were observed under microscope and representative images captured.

### Statistical Analysis

All the experiments were carried out with at least three replicate measurements (intra experimental) and two independent analysis (duplicate analysis) Average values were calculated and the results expressed as mean ± SEM. The statistical significance was performed using one-way ANOVA, followed by Tukey’s *post hoc* test using SPSS software 20 (SPSS, Cary, NC, United States); *P* < 0.05 was considered significant.

## Results

Activation of Nrf2 by naturally occurring antioxidants such as naringenin is one of the potentially viable strategies to treat diseases where Nrf2 plays a key role in disease management.

### Naringenin Upregulated the Expression of Nrf2 in MIN6 Cells

**FIGURE 2:**
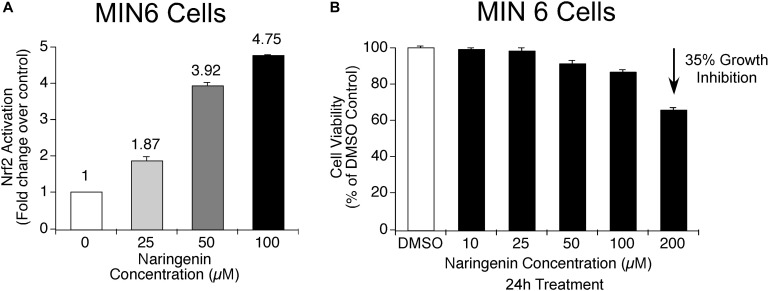
Naringenin activates Nrf2 in MIN6 cells. **(A)** Nrf2 activation potential of naringenin was measured using Nrf2-Keap1 complementation system at 25.0 μM, 50.0 and 100 μM concentrations in MIN6 cells (Ramkumar et al., 2013). The sensor system identifies the potential of naringenin to promote the dissociation of the Nrf2-Keap1 complex. A drop in luciferase signal is inversely proportional to Nrf2 activation. A concentration-dependent activation of Nrf2 by naringenin was observed indicating the dissociation of the complex. **(B)** The concentrations of naringenin used to treat MIN6 cells found not toxic. In order to determine whether the concentration of naringenin used to treat MIN6 cells had any impact on the viability of cells, an MTT assay was carried out as detailed in experimental methods section. Data represents mean ± SEM of three independent experiments. Statistical analysis was performed by one-way ANOVA (*P* < 0.05), followed by Tukey’s *post hoc* test. ^∗^Significant compared with untreated control.

In order to determine whether naringenin has the potential to activate master regulator of antioxidant proteins, i.e., Nrf2, the level of activated Nrf2 was measured using a complementation system in MIN6 cells (Ramkumar et al., 2013). MIN6 cells are known to contain very low endogenous antioxidant machinery compared to many other cells and express minimal Nrf2 (Grankvist et al., 1981). In addition, MIN6 cells are very sensitive to changes in antioxidant levels in the surroundings, hence, they respond quickly to various stress signals (Cheng et al., 2012). Therefore, MIN6 cells are the best suitable cells to study the effect of ROS inducers as well as to determine the ability of antioxidant molecules to trigger Nrf2 transcription factors (Elango et al., 2016). Experimentally, the ability of naringenin (25, 50, and 100 μM for 24 h) to dissociate the Nrf2-Keap1 complex was measured using the Nrf2-Keap1 complementation system in MIN6 cells (Elango et al., 2016). After treatment with naringenin, MIN6 cells were harvested and luciferase activity measured using a luminometer. The developed sensor system detects the potential of naringenin to stimulate the Nrf2-Keap1 complex dissociation. A fall in luciferase signal is inversely proportional to the activation of Nrf2 (Ramkumar et al., 2013). The data showed a dose-dependent increase in the Nrf2 activity (Figure 2A). For example, at 50 and 100 μM concentration of naringenin an about 3.9- and 4.8- fold increase, respectively, was observed (Figure 2A). At these concentrations of naringenin showed no cytotoxic symptoms (Figure 2B).

### Naringenin Promoted the Translocation of Nrf2 to Nucleus and Increased the Expression of Nrf2 Target Genes, NQO1 and GST in MIN6 Cells

**FIGURE 3:**
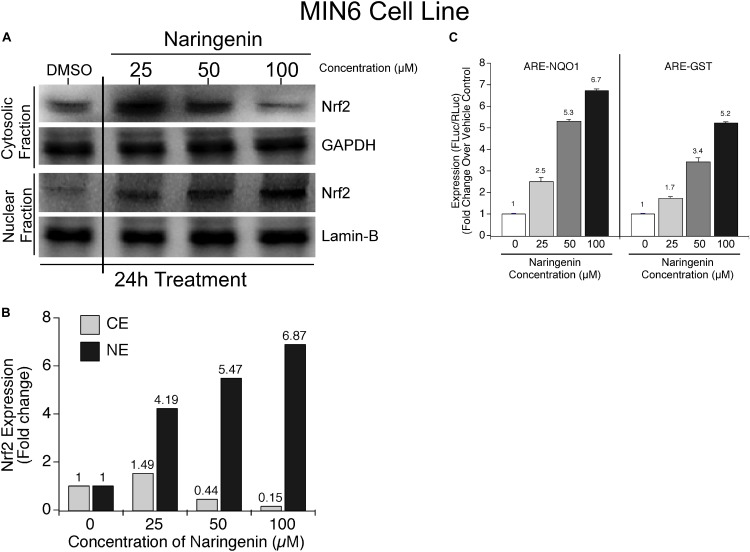
Naringenin induced nuclear translocation of Nrf2 promoted the transcription of NQO1 and GST in MIN6 cells. **(A,B)** *Naringenin-induced Nrf2 is predominantly localized in the nucleus of MIN6 cells.* Analysis of the protein lysates collected from control and naringenin treated cells by western blotting showed a significant increase in nuclear Nrf2 upon exposure of MIN6 cells to 50 and 100 μM naringenin. **(C)** *Naringenin augmented the ARE-driven expression of Nrf2 target genes, NQO1, and GST, in MIN6 cells.* MIN6 cells were transfected with ARE-hNQO1 or ARE-GST luciferase constructs as detailed in Section “Materials and Methods” (Elango et al., 2016). Transfected cells were treated with 25, 50, and 100 μM naringenin for 24 h and luciferase assay performed. Luciferase activity represented as fold induction relative to control cells. Data showed was the average of 3 independent experiments with mean ± SEM. Statistical analysis was performed by one-way ANOVA (*P* < 0.05), followed by Tukey’s *post hoc* test. ^∗^Significant compared with untreated control, ^#^Significant compared with respective control; *P* < 0.05.

Since naringenin exhibited the ability to activate Nrf2 in complementation assay, next, we have determined the efficacy of naringenin to promote the translocation of Nrf2 by isolating the nuclear and cytoplasmic fractions of MIN6 cells exposed to 25, 50, and 100 μM naringenin for 24 h. The amount of Nrf2 in each fraction was measured using western blotting and normalized to the respective loading controls (Elango et al., 2016). The data showed a concentration-dependent increase in the nuclear Nrf2 with a concomitant decrease in the cytosolic fraction (Figure 3A,B).

Next, to further determine whether the translocated Nrf2 is functionally active and elevates the expression of target genes NQO1 and GST, a luciferase-based reporter assay was carried out using ARE-LUC-NQO1 and ARE-LUC-GST constructs (Ramkumar et al., 2013). MIN6 cells were transfected with luciferase-expressing ARE-NQO1 and ARE-GST constructs and the transfected cells treated with 25, 50, and 100 μM naringenin for 24 h. Cell lysates were collected and analyzed for the luciferase activity. Analysis of the data revealed a dose-dependent increase in the luciferase activity in cells harboring NQO1 and GST constructs indicating that naringenin-induced Nrf2 is functionally active and promotes the expression of its target genes NQO1 and GST (Figure 3C).

### Streptozotocin Induced Apoptosis in MIN6 Pancreatic β-Cells by Elevating Caspase-3 Expression and Increasing ROS Levels

Streptozotocin (STZ) is a naturally occurring toxin produced by *Streptomyces achromogenes.* STZ is known to induces diabetes by specifically destroying pancreatic β-cells (Schnedl et al., 1994). Mechanistically STZ inhibits DNA synthesis, induce the generation of free radicals and nitric oxide in pancreatic β-cells thereby promotes pancreatic β-cells death (Schnedl et al., 1994). As a result of pancreatic β-cells destruction, the animals exposed to STZ develop diabetes (Eleazu et al., 2013). Since naringenin potentially upregulates the expression of functionally active Nrf2 in MIN6 cells, its’ administration is predicted to reduce the cellular oxidative stress thereby protect β-cells from undergoing apoptosis (induced by STZ). To test this hypothesis, first, the levels of apoptosis induced by STZ in MIN6 cells were measured and, next, the ability of naringenin to protect MIN6 cells from STZ-induced apoptosis was quantitated.

#### Addition of STZ Induced ROS Production in MIN6 Cells

**FIGURE 4:**
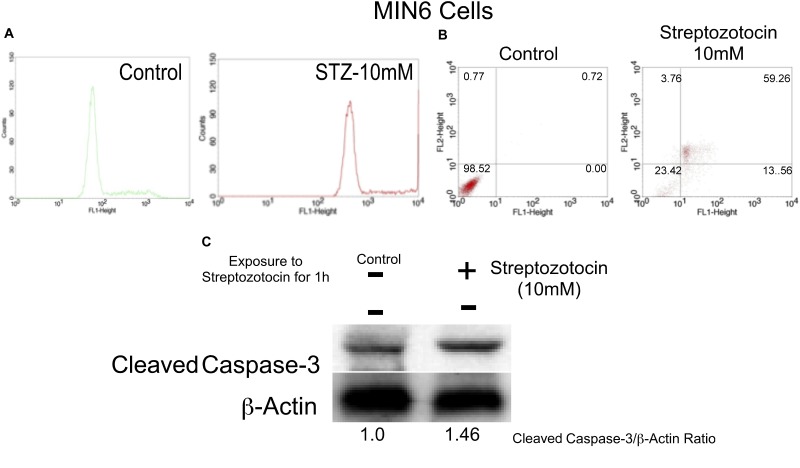
Streptozotocin induced apoptosis in MIN6 cells by elevating caspase-3 and ROS levels. **(A)** *STZ treatment increased the levels of intracellular ROS in MIN6 cells.* MIN6 cells were treated with STZ (10 mM) for 1 h and then H_2_DCFDA (20 μM) was added. The cells were further incubated for 30 min at 37°C and subjected to H_2_DCFDA-fluorescence analysis. The fluorescent peak in STZ-treated cells shifted compared to the peak in control treated cells indicating the production of high levels of ROS. **(B)** *STZ induced apoptosis in MIN6 cells*. First, MIN6 cells were treated with STZ (10 mM) for 1 h. Next, cells were incubated with Annexin-V/PI and analyzed by Flow Cytometry. The scatter plots were divided into four quadrants: UL (Upper Left quadrant representing Annexin-V negative, PI positive), UR (Upper Right quadrant representing both Annexin V and PI positive), LL (Lower Left quadrant representing both Annexin-V and PI negative), LR (Lower Right quadrant representing Annexin-V positive and PI negative). The results showed that STZ treatment resulted in apoptotic cell death. **(C)** *STZ enhanced the expression of caspase-3 in MIN6 cells*. Level of caspase-3 expression in control and STZ treated cells was determined using western blotting. First, MIN6 cells were treated with STZ (10 mM) for 1 h, and cells lysed, centrifuged, and analyzed for total protein using Bradford assay. Next, quantitated protein was separated on polyacrylamide gels and transferred to nitrocellulose membranes. The level of caspase-3 was detected using a primary antibody specific for caspase-3. Analysis of the results showed that STZ treatment increased the caspase-3 expressions by 1.46 fold, compared with the control treatment (1.0).

In order to elucidate the effect of STZ on ROS levels, MIN6 cells were treated with 10 mM STZ for 1 h and the cells subjected to FACS analysis to estimate H_2_DCFDA-fluorescence (Kang et al., 2011). Principally, the non-fluorescent H_2_DCFDA undergoes de-esterification in the cells and form a highly fluorescent 2′,7′-dichlorofluorescein (DCF) upon oxidation (Cohn et al., 2008). The resulted DCF was detected using a fluorescence analyzer. Analysis of the data showed increased intracellular ROS levels with a significant shift of the peak (shift in relative fluorescence intensity) in MIN6 cells (Figure 4A). A significant shift in the fluorescent peak was observed upon treatment of MIN6 cells with 10mM STZ compared to control cells treated with DMSO (Figure 4A). Therefore, the addition of STZ to MIN6 cells triggered ROS generation and oxidative stress.

#### Addition of STZ to MIN6 Cells Increased Apoptosis

The ability of STZ to induce apoptosis in MIN6 cells was estimated by staining with Annexin-V and PI. The ratio of apoptotic and necrotic cells was determined and the results represented in Figure 4B. Analysis of the results showed a significant increase in the number of apoptotic cells upon treatment of MIN6 cells with STZ. Compared to the control treatment, the percentage of early apoptotic cells increased to 13.56%, late apoptotic cells increased to 59.26%, and necrotic cells increased to 3.76% (Figure 4B). Taken together, STZ significantly induced apoptosis in MIN6 cells.

#### STZ Induced Caspase-3 Expression in MIN6 Cells

Apoptosis induced by STZ was further verified by measuring the caspase-3 expression using western blot analysis (Porter and Jänicke, 1999). MIN6 cells were exposed to 10 mM of STZ for 1 h. After treatment, cells were washed with ice-cold PBS, lysed, centrifuged, and analyzed for total protein by the Bradford assay. Samples containing 30 μg of total protein were assayed for caspase-3 expression using western blotting. The data showed a significant increase in the caspase-3 expression (1.46 fold), compared to control treatment (1.0) (Figure 4C). Therefore, it is concluded that STZ is a good inducer of apoptosis in MIN6 cells.

### Naringenin Protected MIN6 Cells From Streptozotocin-Induced Apoptotic Cell Death by Upregulating Nrf2

#### Naringenin Protected MIN6 Cells From STZ-Induced Cell Death

**FIGURE 5:**
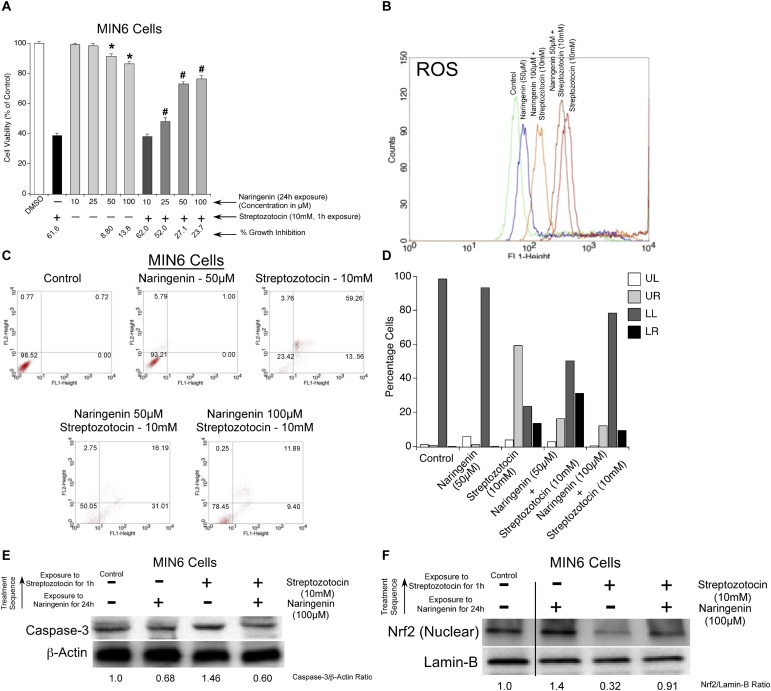
Naringenin protected MIN6 cells from streptozotocin induced apoptotic cell death. **(A)** *Naringenin protected MIN6 cells from STZ-induced cytotoxicity.* To determine the protective effect of naringenin on STZ-induced cytotoxicity, a cell viability assay was carried out using MTT reagent. MIN6 cells were treated with naringenin (0–100 μM) alone, and naringenin for 24 h followed by STZ (10 mM) for 1 h. Naringenin-pretreated cells showed increased viability compared to the STZ (10 mM) alone treated cells. Data represented is the mean ± SEM of three separate experiments. Statistical analysis was performed by one-way ANOVA (*P* < 0.05), followed by Tukey’s *post hoc* test. ^∗^Significant compared with DMSO-treated control, ^#^Significant compared with STZ treated group; *P* < 0.05. **(B)** *Naringenin inhibited the STZ-treatment induced ROS accumulation in MIN6 cells:* Intracellular ROS levels were estimated in MIN6 cells by 2′,7′-dichlorodihydrofluorescein diacetate (H_2_DCFDA) staining followed by analysis of stained cells using FACS. MIN6 cells were pretreated with naringenin (0–100 μM) for 24 h, followed by exposing the cells to 10 mM STZ for 1 h and then subjected to H_2_DCFDA-fluorescence analysis. A greater shift in the peak position implies the higher amount of DCF and greater ROS generation. **(C,D)** *Naringenin could mitigate the apoptosis inducing effect of STZ in MIN6 cells:* MIN6 cells were, first, pretreated with naringenin (0–100 μM) for 24 h, followed by STZ treatment (10 mM) for 1 h. Analysis of Annexin and PI stained cell showed decrease in early apoptotic cells with naringenin (100 μM) treatment. **(E)** *STZ-treatment induced caspase-3 was reduced by pretreatment with naringenin in MIN6 cells*. MIN6 cells were pretreated with naringenin (0–100 μM) for 24 h, followed by exposure to 10 mM STZ for 1 h. Cell lysates were collected and analyzed by SDS-PAGE followed by Western blotting to detect caspase-3 expression using specific antibody. Analysis of the blots showed a significant increase in the caspase 3 (1.46 fold) upon exposure to STZ. **(F)** *Naringenin upregulated Nrf2 in STZ-treated MIN6 cells.* To check whether naringenin treatment increased the Nrf2 level in MIN6 cells incubated with STZ, the nuclear fractions were collected and analyzed for Nrf2 expression using western blotting. The authenticity of nuclear fraction was confirmed by probing with laminin-B. A significant decrease in nuclear Nrf2 was observed in STZ treated cells.

To check whether antioxidant naringenin could protect MIN6 cells from STZ-induced cell death, the viability of MIN6 cells was estimated using MTT assay. Experimentally, MIN6 cells were first pre-treated with non-cytotoxic concentrations of naringenin (0–100 μM) for 24 h and then exposed to 10 mM STZ for 1 h. Cell viability was then measured using the MTT assay and the percentage growth inhibition calculated by comparing with vehicle-treated cells (Mosmann, 1983). Cells pre-treated with naringenin showed increased viability of 47.95, 72.88, and 76.24% at 25, 50, and 100 μM concentrations, respectively, compared to the 10 mM STZ treated cells (38.43%) (Figure 5A).

#### Treatment With Naringenin Reduced STZ-Induced ROS Production in MIN6 Cells

Reactive oxygen species play a significant role in the pathogenesis of STZ-induced diabetes (Asmat et al., 2016). Therefore, to determine the potency of naringenin to mitigate the levels of STZ-induced ROS, MIN6 cells were first treated with naringenin (0–100 μM) for 24 h, followed by exposing the cells to 10 mM STZ treatment for 1 h. These cells were subjected to H_2_DCFDA-fluorescence analysis. Mechanistically, the non-fluorescent H_2_DCFDA gets de-esterified intra-cellularly to produce fluorescent 2′,7′-dichlorofluorescein (DCF) (Cohn et al., 2008). Compared to control untreated naringenin, exposure to STZ for 1 h significantly induced ROS production, i.e., the fluorescent peak in STZ-treated cells shifted toward the right (Figure 5B). However, STZ-induced ROS production was reduced by naringenin treatment in a dose-dependent manner (Figure 5B). The shift in peak, toward untreated control peak, upon naringenin treatment (50 and 100 μM) indicates a diminished ROS level as shown by a change in the fluorescence intensity when compared to STZ treatment (Figure 5B). These results indicate that naringenin inhibits ROS generation thereby minimizing the damage caused to MIN6 cells.

#### Prior Exposure of MIN6 Cells to Naringenin Could Decrease the Apoptosis Inducing Effect of STZ

To assess the cytoprotective effect of naringenin against STZ treatment-induced toxicity in MIN6 cells, the levels of apoptosis was estimated by FACS using Annexin-V/PI staining (Hingorani et al., 2011). Flipping of phosphatidylserine of the plasma membrane from the inner surface to the outer surface is an early event in apoptosis (Hingorani et al., 2011). Annexin-V binds to the phosphatidylserine, hence, labeled Annexin-V helps in the detection of cells undergoing apoptosis (Hingorani et al., 2011). Propidium iodide is used in combination with labeled Annexin-V, to measure the cell membrane integrity as the propidium iodide is impermeable if the cells are viable, but can enter the cells that are undergoing apoptosis or necrosis (Cohn et al., 2008). Experimentally, MIN6 cells were pretreated with naringenin (0–100 μM) for 24 h, followed by treatment with 10 mM STZ treatment for 1 h. Next, cells were treated with Annexin-V and Propidium Iodide and analyzed by flow cytometry. The collected data was analyzed using cell quest pro-software. Analysis of the results showed no major cell death with 50 μM naringenin treatment (Figure 5C,D). However, treatment with STZ (10 mM for 1 h) induced apoptosis in MIN6 cells (Figure 5C,D). A significant increase in early apoptotic cells (13.56%) and late apoptotic cells (59.26%) along with 3.76% necrotic cells was observed in STZ treated cells. Prior treatment with 50 and 100 μM naringenin could reduce the apoptosis compared to STZ treated cells (Figure 5C,D). For instance, 100 μM naringenin treatment reduced the percentage of early apoptotic cells to 9.40% from 13.56% (10 mM STZ treated), late apoptotic cells reduced to 11.89% from 59.26% (10mM STZ treated) and necrotic cells reduced to 0.25% from 3.76% (10 mM STZ treated). Taken together, this data showed the ability of naringenin to mitigate the effect of STZ-induced apoptosis in MIN6 cells.

#### Naringenin Decreased the Caspase-3 Expression in STZ-Treated MIN6 Cells

To further determine the anti-apoptotic effects of naringenin, MIN6 cells were pretreated with naringenin (0–100 μM) for 24 h, followed by exposure to 10 mM STZ treatment for 1 h. Clear cell lysates were collected by centrifugation and total protein quantitated by the Bradford method (Kruger, 2009). About 30 μg of total protein was used to study the expression of caspase-3 by probing with a specific antibody. Exposure of MIN6 cells to STZ caused an increase in caspase-3 expression compared to control cells without treatment. Prior exposure of cells to naringenin decreased the STZ-induced caspase-3 expression (Figure 5E).

#### Cytoprotective Effect of Naringenin Is Mediated by the Upregulation of Nrf2 in MIN6 Cells

Since naringenin exhibited the activation of cytoprotective Nrf2, next, the effect of naringenin against STZ induced toxicity was estimated. In order to study this hypothesis, first, MIN6 cells were treated with naringenin and cytosolic and nuclear fractions separated using a commercially available nuclear extraction kit (Pierce NE-PER^®^) as per the manufacturer’s instructions (Pierce, Rockford, IL, United States). The total protein content in the separated fractions was estimated using the Bradford method and level of Nrf2 expression analyzed using western blotting. Analysis of the data showed a significant increase in the Nrf2 level upon treatment of MIN6 cells with 100 μM naringenin, which protected MIN6 cells against STZ-induced toxicity when compared to untreated and STZ-treated cells (Figure 5F).

### Oral Administration of Naringenin Reduced the STZ-Induced Fasting Blood Glucose Levels, Improved Glucose Tolerance (IPGTT) and Response to Insulin in STZ-Treated Mice

**FIGURE 6:**
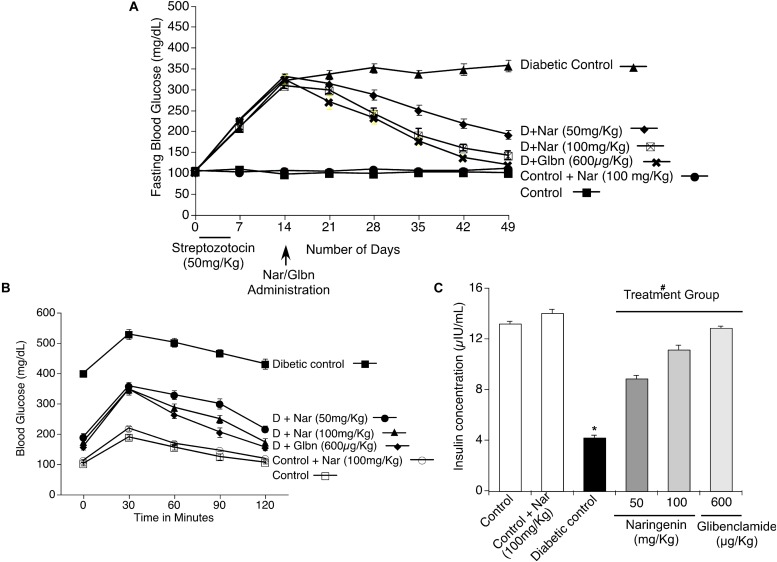
Effects of naringenin on STZ induced hyperglycemia, serum insulin level and intra peritoneal glucose tolerance test (IPGTT) in control and experimental mice. **(A)** *Naringenin administration could decrease the STZ-induced glucose levels in mice.* Streptozotocin, a glucopyranose derivative of N-methyl-N-nitrosourea, is a unique diabetogenic compound known to induce diabetes in mice. Administration of 50 and 100 mg/kg b.w. of naringenin showed a significant dose-dependent reduction in blood glucose level compared to STZ control group. Similarly, 600 μg/kg b.w. glibenclamide showed a marked reduction in blood glucose level compared to STZ control group. **(B)** *Naringenin could improve the response of mice to glucose load in intra peritoneal glucose tolerance test (IPGTT).* The anti-hyperglycemic effect of naringenin was conformed by assessing the IPGTT in STZ induced diabetic mice. **(C)** *Naringenin could improve the serum insulin levels in STZ-induced diabetic mice*: Data are mean ± SE from six mice in each group. Statistical analysis was performed by one-way ANOVA (*P* < 0.05), followed by Tukey *post hoc* test. ^∗^STZ control mice were compared with control mice. ^#^STZ + NAR (50 and 100 mg/kg b.w.) and STZ + GLC (600 μg/kg b.w.) treated mice were compared with STZ control mice; *P* < 0.05.

Since naringenin showed a better cytoprotective effect in the *in vitro* experiments, next, its ability to decrease the complications of diabetes, especially blood glucose, was tested in preclinical animal models. Experimentally, first, diabetes was induced by MLD-STZ administration (i.p.; 50 mg/kg body weight/day) for 5 days. As shown in Figure 6A, all mice showed hyperglycemia with a serum glucose level of 357 ± 13.6 mg/dl in comparison with control mice exposed to vehicle 0.5% CMC (99.5 ± 4.9 mg/dl). Oral administration of naringenin for 45 days caused a significant (*p* < 0.05) dose-dependent (50 and 100 mg/kg b.w.) reduction in blood glucose levels in STZ mice. For example, blood glucose levels of STZ mice were reduced from 357 ± 13.6 mg/dl to 141.25 ± 10.6 mg/dl after administration of naringenin at the dose of 100 mg/kg b.w. These results were comparable with positive control glibenclamide at 600 μg/kg b.w (Figure 6A). However, naringenin (100 mg/kg b.w.) treated control mice did not show any statistically significant (*p* < 0.05) difference when compared to that of control vehicle-treated mice (Figure 6A). In summary, naringenin could reduce the STZ-induced hyperglycemia in mice.

#### Oral Administration of Naringenin Could Improve the Glucose Tolerance in STZ-Treated Mice

In order to assess whether naringenin administration helps to improve the ability of mice to process ingested glucose, IPGTT was carried out as detailed in methods section (Goren et al., 2004). After 45 days of treatment with naringenin, mice fasted for 12 h and saline glucose solution at 1 g/kg b.w. injected by intraperitoneal route (Goren et al., 2004). Glucose levels were measured in the blood collected from the tail vein at 30, 60, 90, and 120 min after the glucose load. All the groups showed an initial sharp increase of glucose up to 30 min. Administration of 50 and 100 mg/kg b.w. of naringenin and 600 μg/kg b.w. of glibenclamide (positive control) showed a progressive decrease in the glucose level from 60 to 120 min that was comparable with the control 0.5% CMC treated group (Figure 6B). But, in the STZ group, the blood glucose levels remained high even after 120 min (430.75 ± 16.3 mg/dl) (Figure 6B). Therefore, the glucose tolerance was significantly improved by naringenin treatment. Control animals administered with 100 mg/kg b.w. naringenin showed a normal pattern of glucose tolerance (Figure 6B).

#### Oral Administration of Naringenin Improved Insulin Secretion in STZ-Treated Mice

In order to evaluate β-cell function in terms of insulin release, serum insulin level was measured after 45 days of treatment. STZ mice showed a significant (*p* < 0.05) decrease in serum insulin (from 13.16 ± 0.2 μIU/ml to 4.1 ± 0.2 μIU/ml) compared to vehicle-treated control mice (Figure 6C). Oral administration of naringenin for a period of 45 days, dose-dependently increased the levels of insulin in STZ treated mice. Administration of 50 mg/kg b.w. and 100 mg/kg b.w. naringenin increased the insulin levels to 8.8 ± 0.2 μIU/ml and 11.1 ± 0.4 μIU/ml, respectively (Figure 6C). The positive control 600 μg/kg b.w. glibenclamide augmented the insulin levels up to 12.8 ± 0.1 μIU/ml, which is similar to control animals (Figure 6C). Treatment of control animals (exposed to vehicle 0.5% CMC) with 100 mg/kg b.w. naringenin did not change insulin levels (Figure 6C). The increased insulin level with naringenin treatment could be due to the insulinotropic effect, which helps in the protection and/or regeneration of β-cells so that they produce sufficient insulin.

### Oral Administration of Naringenin Restored the STZ-Induced Altered Lipid Profile in Mice

The anti-hyperlipidemic efficacy of naringenin was investigated in STZ treated mice by measuring the levels of lipids in serum (de Almeida et al., 2012) (Table 2). STZ administered animals showed significantly (*P* < 0.05) increased serum (a) total cholesterol (TC) from 75.03 ± 6.2 mg/dl to 181 ± 2.0 mg/dl, (b) LDL from 16.6 ± 8.1 mg/dl to 127.2 ± 2.2 mg/dl, (c) VLDL from 10.26 ± 0.5 mg/dl to 27.06 ± 1.3 mg/dl, (d) Triglycerides (TG) from 51.33 ± 2.5 mg/dl to 135.3 ± 6.5 mg/dl, (e) Phospholipids from 82.33 ± 4.8 mg/dl to 114.5 ± 7.8 mg/dl, (f) Free fatty acids (FFA) from 2.43 ± 0.4 mg/dl to 5.56 ± 0.2 mg/dl, and significantly decreased HDL level from 48.16 ± 3.1 mg/dl to 26.7 ± 1.24mg/dl (Table 2). Oral administration of naringenin (50 and 100 mg/kg b.w.) showed a significant (P < 0.05) reduction in TC, LDL, VLDL, TG, Phospholipids, and FFA. In addition, a significant (*p* < 0.05) increase in the concentration of HDL compared to STZ controls was noted (Table 2). For instance, TC was decreased from 181 ± 2.0 mg/dl to 106.6 ± 4.9 mg/dl. Likewise serum LDL was reduced from 127.2 ± 2.2 mg/dl to 47.56 ± 6.1 mg/dl and VLDL from 27.06 ± 1.3 mg/dl to 17.33 ± 1.1 mg/dl. Similarly, while triglyceride content was reduced from 135.3 ± 6.5 mg/dl to 86.66 ± 5.5 mg/dl the phospholipid level decreased from 114.5 ± 7.8 mg/dl to 87.05 ± 7.2 mg/dl upon exposure to naringenin. Furthermore, whereas free fatty acids showed a significant decrease from 5.56 ± 0.2 mg/dl to 2.76 ± 0.2 mg/dl, the level of HDL content increased from 26.7 ± 1.24 mg/dl to 41.76 ± 1.2 mg/dl with naringenin administration (Table 2). Administration of the positive control glibenclamide (600 μg/kg b.w.) to STZ-induced diabetic mice has restored the lipid profile to normal levels (Table 2). The observed anti-hyperlipidemic effect of naringenin could be due to either its insulin stimulatory effect on pancreatic β-cells or due to decreased cholesterogenesis and fatty acid synthesis.

**Table 2:** Effect of naringenin on serum lipid profile in control and STZ-treated mice.

Groups	Serum Lipid Profile						
	Total Cholesterol (mg/dl)	Triglycerides (mg/dl)	HDL-C (mg/dl)	LDL-C (mg/dl)	VLDL-C (mg/dl)	Phospholipids (mg/dl)	Free Fatty Acids (mg/dl)
Control	75.03 ± 6.2	51.33 ± 2.5	48.16 ± 3.1	16.6 ± 8.1	10.26 ± 0.5	82.33 ± 4.8	2.43 ± 0.4
NAR control (100 mg/kg)	73.46 ± 5.8	48.33 ± 3.5	50.73 ± 2.6	13.0 ± 8.17	9.66 ± 0.7	77.90 ± 3.7	1.93 ± 0.5
STZ control	181 ± 2.0^∗^	135.3 ± 6.5^∗^	26.7 ± 1.24^∗^	127.2 ± 2.2^∗^	27.06 ± 1.3^∗^	114.5 ± 7.8^∗^	5.56 ± 0.2^∗^
STZ + NAR (50 mg/kg)	138.4 ± 1.8^#^	111.6 ± 6.1^#^	37.33 ± 3.9^#^	78.76 ± 2.7^#^	22.33 ± 1.2^#^	94.15 ± 5.6^#^	3.16 ± 0.3^#^
STZ + NAR (100 mg/kg)	106.6 ± 4.9^#^	86.66 ± 5.5^#^	41.76 ± 1.2^#^	47.56 ± 6.1^#^	17.33 ± 1.1^#^	87.05 ± 7.2^#^	2.76 ± 0.2^#^
STZ + GLC (600 μg/kg)	95.7 ± 2.8^#^	72.33 ± 4.1^#^	45.2 ± 3.79^#^	36.03 ± 5.8^#^	14.46 ± 0.8^#^	85.07 ± 6.8^#^	2.53 ± 0.1^#^

*HDL-C, high-density lipoprotein-cholesterol; LDL-C, low-density lipoprotein-cholesterol; VLDL-C, very low-density lipoprotein-cholesterol. Injection of STZ to mice not only increased the levels of blood glucose but also elevated the serum lipids as evidenced by high TG, TC, LDL, VLDL, phospholipids, free fatty acids and reduced HDL (Krishnakumari et al., 2011). Treating these diabetic animals with naringenin (50 and 100 mg/kg b.w.) for 45 days significantly (p < 0.05) reduced the elevated lipids. The positive control 600 μg/kg b.w. glibenclamide also showed significant reduction in the serum lipids. Data represents mean ± SE from six mice in each group. Statistical analysis was performed by one-way ANOVA (P < 0.05), followed by Tukey’s post hoc test. ^∗^STZ mice were compared with control mice. ^#^STZ + NAR (50 and 100 mg/kg b.w.) and STZ + GLC (600 μg/kg b.w.) treated mice were compared with STZ control mice; P < 0.05.*

### Oral Administration of Naringenin Retarded the Formation of TBARS and Hydroperoxides in STZ-Treated Pancreatic Tissues

Lipid peroxidation and formation of hydroperoxides are key indicators of tissue damage, which occurs due oxidative stress (El-Aal, 2012). Since STZ is known to promote cellular oxidative stress, first, the levels of lipid peroxides were measured by boiling 0.1 ml tissue homogenate with 2.0 ml of TBA:TCA:HCl reagent for 15 min followed by cooling, and centrifuging at 10,000 rpm for 5 min to separate clear supernatant. The levels of TBARS (mM/100 g tissue) were estimated by reading the absorption at 535 nm (Niehaus and Samuelsson, 1968). Table 3 shows a significant increase in the concentration of TBARS in pancreatic tissue from about 2 mM/100 g tissue to ∼4.2 mM/100 g tissue upon STZ treatment. Administration of 50 mg/kg b.w. and 100 mg/kg b.w. naringenin significantly reduced the TBARS in pancreatic tissue to ∼3.6 mM/100 g tissue and ∼2.8 mM/100 g tissue, respectively (Table 3). The positive control 600 μg/kg b.w. glibenclamide reduced the TBARS levels to ∼2.5 mM/100 g tissue (Table 3). Treatment of control animals (exposed to vehicle 0.5% CMC) with 100 mg/kg b.w. naringenin did not change TBARS levels (Table 3).

**Table 3:** Changes in the levels of TBARS and hydroperoxides in pancreatic tissues of control and experimental mice.

Groups	TBARS (mM/100 g tissue)	Hydroperoxides (mM/100 g tissue)
Control	2.16 ± 0.15	16.40 ± 0.18
NAR control (100 mg/kg)	1.97 ± 0.09	16.12 ± 0.19
STZ control	4.22 ± 0.20^∗^	22.96 ± 1.19^∗^
STZ + NAR (50 mg/kg)	3.68 ± 0.07^#^	20.67 ± 0.49^#^
STZ + NAR (100 mg/kg)	2.82 ± 0.09^#^	17.66 ± 0.98^#^
STZ + GLC (600 μg/kg)	2.56 ± 0.20^#^	16.41 ± 0.20^#^

*The levels of TBARS and hydroperoxide concentration were determined using colorimetric assays as described in materials and methods section (Niehaus and Samuelsson, 1968). Analysis of the results showed a significant increase in the levels of TBARS and hydroperoxides (p < 0.05) upon administering STZ to mice. Oral administration of 50 and 100 mg/kg b.w. of naringenin and 600 μg/kg b.w. of glibenclamide (positive control) showed a significant decrease in TBARS and hydroperoxides levels. Data represents mean ± SE from six mice in each group. Statistical analysis was performed by one-way ANOVA (P < 0.05), followed by Tukey’s post hoc test. ^∗^STZ control mice were compared with control mice. ^#^STZ + NAR (50 and 100 mg/kg b.w.) and STZ + GLC (600 μg/kg b.w.) treated mice were compared with STZ control mice; P < 0.05.*

To estimate the concentration of hydroperoxides, 0.2 ml of tissue homogenate was incubated with 1.8 ml of Fox reagent at room temperature for 30 min and the absorbance measured at 560 nm (Jiang et al., 1992). The results were expressed as mM hydroperoxides produced per 100 g tissues. The STZ-treatment significantly elevated the tissue pancreatic hydroperoxides from 16 mM/100 g tissue to ∼23 mM/100 g tissue (Table 3). Elevated pancreatic hydroperoxides were brought down to about ∼20 mM/100 g tissue and ∼17 mM/100 g tissue, respectively upon 50 mg/kg b.w. and 100 mg/kg b.w. naringenin administration (Table 3). Oral administration of glibenclamide (600 μg/kg b.w.) decreased hydroperoxides levels (to ∼16 mM/100 g) that are similar to control animals (Table 3). Treatment of control animals with 100 mg/kg b.w. naringenin did not change hydroperoxides levels (Table 3).

### Oral Administration of Naringenin Enhanced the Expression of Nrf2 and Thereby Increased the Activities of SOD, CAT, GPX, GST Enzymes and the Levels of GSH in the Pancreatic Tissues of STZ-Treated Mice

**FIGURE 7:**
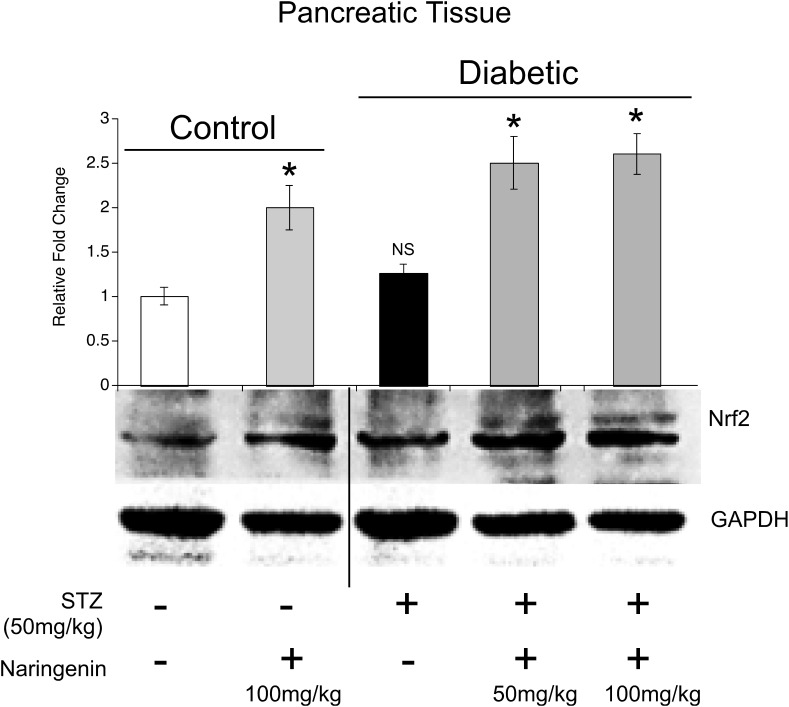
Naringenin upregulated the expression of Nrf2 in pancreatic tissue: Pancreatic tissue lysates were generated and analyzed by western blotting to detect Nrf2 and the loading control GAPDH. Analysis of the data showed a significant increase in the Nrf2 expression in tissues collected from animals treated with naringenin compared to vehicle and streptozotocin administered ones. Interestingly, a slight non-significant increase in Nrf2 was observed even in the STZ administered animals. ^∗^Naringenin (100 mg/kg b.w.) and STZ C NAR (50 and 100 mg/kg b.w.) treated mice were compared with vehicle control and STZ control mice; *P* < 0.05 (One-way ANOVA).

Since, *in vitro* studies have demonstrated the Nrf2 activation potential of naringenin, we have hypothesized that the cytoprotective effects of naringenin might be mediated through similar mechanisms even in mice. Therefore, protein lysates were collected from the pancreatic tissues of control and experimental animals and subjected for western blotting. Analysis of the data showed an about 2.5-fold increase in the Nrf2 expression compared to control or STZ treated mice (Figure 7). However, interestingly, a slight non-significant increase in the Nrf2 expression (compared to control mice) was observed in STZ treated mice (Figure 7). It is well known that Nrf2 regulates the cellular oxidative stress by increasing the expression of its target genes NQO1, SOD, CAT, GPX, and GSH transferase (Jung and Kwak, 2010). Therefore, we have measured the activities of these Nrf2 target genes in the pancreatic tissue (Table 4). SOD is an enzyme, which catalyzes the dis-mutation of superoxide radicals to H_2_O_2_ thus decreasing the possibility of superoxide anion interacting with nitric oxide that forms reactive peroxynitrite (Fukai and Ushio-Fukai, 2011). The SOD activity in the tissues was assayed according to the method developed by Kakkar et al. (1984). The reaction was initiated by addition of NADH. After incubation for 90 s, the glacial acetic acid was added to stop the reaction and the colored complex formed was extracted into butanol layer and measured at 560 nm. The SOD activity was significantly (*p* < 0.05) decreased in pancreatic tissue from about ∼6.4 units/min/mg protein to ∼2.1 units/min/mg protein upon STZ injection (Table 4). Administration of 50 mg/kg b.w. and 100 mg/kg b.w. naringenin significantly augmented the SOD levels in pancreatic tissue to ∼4.5 units/min/mg protein and ∼5.5 units/min/mg protein, respectively (Table 4). The positive control 600 μg/kg b.w. glibenclamide augmented the SOD levels to ∼6.1 units/min/mg protein (Table 4). Treatment of control animals with 100 mg/kg b.w. naringenin did not change SOD levels (Table 4).

**Table 4:** Effect of naringenin on the activities of SOD, CAT, GPX, GST, and GSH in the pancreatic tissues of control and experimental mice.

Groups	SOD (Units/mg Protein)	CAT (Units/mg Protein)	GPX (Units/mg Protein)	GST (Units/mg Protein)	GSH (mg/100 g tissue)
Control	6.41 ± 0.20	18.76 ± 1.11	5.46 ± 0.20	4.175 ± 0.2	11.28 ± 0.12
NAR control (100 mg/kg)	6.52 ± 0.37	18.90 ± 0.75	5.64 ± 0.35	4.332 ± 0.2	12.03 ± 0.37
STZ control	2.12 ± 0.10^∗^	7.83 ± 0.56^∗^	2.81 ± 0.19^∗^	1.392 ± 0.1^∗^	6.18 ± 0.133^∗^
STZ + NAR (50 mg/kg)	4.59 ± 0.11^#^	13.82 ± 0.99^#^	4.83 ± 0.07^#^	3.007 ± 0.1^#^	8.60 ± 0.385^#^
STZ + NAR (100 mg/kg)	5.56 ± 0.16^#^	18.05 ± 1.01^#^	5.47 ± 0.12^#^	3.312 ± 0.1^#^	10.73 ± 0.46^#^
STZ + GLC (600 μg/kg)	6.16 ± 0.28^#^	18.54 ± 1.33^#^	5.83 ± 0.16^#^	3.957 ± 0.1^#^	11.24 ± 0.26^#^

*Persistent hyperglycemia modulates the levels of free radicals, antioxidant enzymes and their substrates (Birben et al., 2012). Administration of STZ to mice showed a significant (p < 0.05) decrease in the levels of antioxidants compared to control group (Table 4). Oral administration of 50 and 100 mg/kg b.w. of naringenin and 600 μg/kg b.w. of glibenclamide (a positive control) showed a significant increase in the antioxidants to near normal levels in pancreatic tissues of control mice (Table 4). Data are expressed as mean ± SE from six mice in each group. Statistical analysis was performed by one-way ANOVA (P < 0.05), followed by Tukey’s post hoc test. ^∗^STZ control mice were compared with control mice. ^#^STZ + NAR (50 and 100 mg/kg b.w.) and STZ + GLC (600 μg/kg b.w.) treated mice were compared with STZ control mice; P < 0.05.*

Catalase is a tetrameric hemin-enzyme, which decomposes hydrogen peroxide into water and molecular oxygen (Kirkman and Gaetani, 1984). The CAT activity (μmoles of H_2_O_2_ consumed/min/mg protein) in the tissues was assayed by the method developed by Sinha (1972). Dichromate is converted to chromic acetate after heating in the presence of hydrogen peroxide with the formation of an intermediate perchromic acid. The produced chromic acetate was measured at 590 nm. The CAT activity significantly decreased in pancreatic tissue from about 18 units in control animals to 7.8 units; upon STZ injection (Table 4). Administration of 50 mg/kg b.w. naringenin and 100 mg/kg b.w. naringenin significantly augmented the CAT activity in pancreatic tissue to ∼13.8 and ∼18.0 units, respectively (Table 4). The positive control 600 μg/kg b.w. glibenclamide significantly elevated the CAT activity, which is similar to the activity of control animals (Table 4). No significant change was observed in mice treated with 100 mg/kg b.w. naringenin in comparison to that of control group administered with 0.5% carboxymethyl cellulose demonstrating that naringenin administration has no major impact on CAT activity (Table 4).

GPX is a selenium-containing peroxidase, which decomposes H_2_O_2_ and lipid peroxides through GSH, as a hydrogen donor, into the water and protects the cells from free radicals (Lubos et al., 2011). The activity of GPX was determined by the method of Rotruck et al. (1973). The GPX activity (μmoles of GSH oxidized/min/mg protein) was significantly decreased in pancreatic tissue from about from 5.4 μmoles in control animals to ∼2.8 μmoles; upon STZ treatment (Table 4). Oral administration of 50 mg/kg b.w. naringenin and 100 mg/kg b.w. naringenin significantly augmented the GPX activity in pancreatic tissue to ∼4.8 and ∼5.4 μmoles; respectively (Table 4). The positive control 600 μg/kg b.w. glibenclamide significantly augmented the GPX activity in pancreatic tissue up to 5.8 μmoles compared to STZ control animals (Table 4). No significant change was observed even in non-diabetic mice treated with naringenin in comparison to that of the control group demonstrating that naringenin administration did not affect the GPX levels (Table 4).

GST is a GSH-dependent cytosolic enzyme, which protects cells from the damage caused by ROS (Hayes and McLELLAN, 1999). In order to determine whether administration of naringenin restored the GST activity, a method developed by Habig et al. (1974) was used and the results expressed as μmoles of CDNB conjugate formed /min/mg protein. Analysis of the data represented in Table 4 showed about 75% decrease in GST activity in the pancreatic tissue, compared to vehicle-treated mice, in STZ injected animals. Oral administration of naringenin (50 mg/kg and 100 mg/kg b.w.) and glibenclamide (600 μg/kg b.w.) significantly (*p* < 0.05) increased the GST activity levels. For example, administration of 100 mg/kg body weight naringenin elevated the pancreatic GST activity to ∼3.3 units (Table 4). Similarly, 600 μg/kg body weight glibenclamide elevated the pancreatic GST activity to ∼3.9 units (Table 4). No significant change in GST activity was observed between vehicle control and 100 mg/kg body weight naringenin-treated non-diabetic mice (Table 4).

GSH is a potent free-radical scavenger and co-substrate for GPX activity (Sies, 1999). Several studies have shown the ability of STZ to decrease cellular GSH levels (Birben et al., 2012). Therefore, to determine whether administration of naringenin could mitigate the effect of STZ-induced GSH depletion effect, pancreatic tissues were collected and processed to estimate GSH content using Ellman’s assay (Ellman, 1959). The data in Table 4 shows a significant decrease in pancreatic tissue GSH levels upon administering mice with STZ (from ∼11.28 mg/100 g tissue to ∼6 mg/100 g tissue). However, when naringenin or glibenclamide was administered the levels had gone up to the ones present in control animals (Table 4). However, no significant changes were observed in non-diabetic mice treated with naringenin in comparison to that of a control group of mice (Table 4).

### Oral Administration of Naringenin Restored the Changes in Carbohydrate Metabolizing Enzymes in STZ-Injected Liver Tissues

#### Oral Administration of Naringenin Enhanced the Activity of Hexokinase and Glucose-6-Phosphate Dehydrogenase

The first step in the glycolysis is the fixation of glucose by phosphorylation into glucose-6-phosphate by Hexokinase enzyme (Guo et al., 2012). Hexokinase functions as a glucose sensor of insulin-producing pancreatic β-cells and regulates the glycogen synthesis and hepatic glucose production (Guo et al., 2012). The hexokinase activity in the liver tissue was determined by the method of Brandstrup et al. (1957). Hexokinase converts D-glucose and ATP to glucose 6-phosphate and ADP. The residual glucose was reacted with an *o*-toluidine reagent to form a green color, which was measured at 640 nm. The hexokinase activity was significantly (*p* < 0.05) reduced in liver tissues of STZ control mice from 87.71 ± 3.4 units/mg protein to 60.04 ± 0.9 units/mg protein (Table 5). Oral administration of 50 mg/kg b.w. and 100 mg/kg b.w. naringenin significantly augmented the activity of hexokinase to 74.12 ± 1.0 units/mg protein and 82.54 ± 3.5 units/mg protein, respectively (Table 5). The positive control 600 μg/kg b.w. glibenclamide increased the activity to 85.06 ± 2.4 units/mg protein (Table 5). However, treatment of control non-diabetic animals with 100 mg/kg b.w. naringenin did not show any difference (Table 5).

**Table 5:** Changes in the activities of key enzymes of carbohydrate metabolism and glycogen in the liver of control and experimental mice.

Groups	Hexokinase (IU^@^/g protein)	G6-PD (10^4^mIU/mg protein)	G6-P (IU^∗^mg protein)	F1,6-BP (IU^$^/mg protein)	Glycogen (mg/100 g Tissue)
Control	87.61 ± 3.4	4.29 ± 3.70	0.178 ± 0.004	0.189 ± 0.025	32.44 ± 2.04
NAR control (100mg/kg)	88.97 ± 0.4	4.47 ± 0.27	0.174 ± 0.020	0.184 ± 0.024	32.61 ± 0.91
STZ control	60.04 ± 0.9^∗^	2.61 ± 0.03^∗^	0.297 ± 0.010^∗^	0.440 ± 0.003^∗^	21.27 ± 1.44^∗^
STZ+NAR (50mg/kg)	74.12 ± 1.0^#^	3.62 ± 0.14^#^	0.255 ± 0.005^#^	0.316 ± 0.015^#^	27.88 ± 1.60^#^
STZ+NAR (100mg/kg)	82.54 ± 3.5^#^	4.16 ± 0.09^#^	0.231 ± 0.010^#^	0.267 ± 0.027^#^	29.88 ± 0.98^#^
STZ+GLC (600μg/kg)	85.06 ± 2.4^#^	4.06 ± 0.16^#^	0.202 ± 0.016^#^	0.223 ± 0.026^#^	28.05 ± 0.30^#^

*^@^IU-moles of glucose phosphorylated per min, ^∗^IU-moles of inorganic phosphate liberated per min, ^$^IU-moles of inorganic phosphate liberated per h. G6-PD, Glucose-6-phosphate dehydrogenase; G6-P, Glucose-6-phophatase; F1,6-BP, Fructose-1,6-bisphosphatase. Diabetes inducing agent Streptozotocin decreased the activities of hexokinase and glucose-6-phosphate dehydrogenase, while promoting the activities of glucose-6-phosphatase and fructose-1, 6-bisphosphatase. Oral administration of naringenin (50 and 100 mg/kg b.w.) and glibenclamide (600 μg/kg b.w.) significantly (p < 0.05) increased the activity of hexokinase and glucose-6-phosphate dehydrogenase in liver of STZ-induced diabetic mice indicating improved utilization of glucose by liver in diabetic mice. The activities of glucose-6-phosphatase and fructose-1, 6-bisphosphatase were decreased leading to low gluconeogenesis. In addition, oral administration of naringenin (50 and 100 mg/kg b.w.) and glibenclamide (600 μg/kg b.w.) significantly (p < 0.05) increased the concentration of liver glycogen. Data are expressed as mean ± SE from six mice in each group. Statistical analysis was performed by one-way ANOVA (P < 0.05), followed by Tukey’s post hoc test. ^∗^Significantly different from control at P < 0.05. ^#^Significantly different from diabetes group at P < 0.05.*

Glucose-6-phosphate dehydrogenase (G6-PD) is the first regulating enzyme of the pentose phosphate pathway of glucose metabolism that produces (a) ribose-5-phosphate; (b) reducing equivalent, NADPH and a variety of sugars with carbon chain length beginning from 3C to 7C (Cappai et al., 2011). A method developed by Ells (1961) was used to measure the G6-PD activity in liver tissue (Ells and Kirkman, 1961). The increase in absorbance when NADP reduced to NADPH was measured. The reduction of NADP occurs due to the transfer of electrons from glucose-6-phosphate to NADP, which is catalyzed by G6-PD. The STZ control animals showed a significant (*p* < 0.05) decrease in the activity of G6-PD in liver tissue (4.29 ± 0.3 units/mg protein VS 2.61 ± 0.1 units/mg protein) (Table 5). Oral administration of 50 mg/kg b.w. naringenin and 100 mg/kg b.w. naringenin significantly augmented the G6-PD activity to 3.62 ± 0.1 units/mg protein and 4.16 ± 0.1 units/mg protein, respectively (Table 5). 600 μg/kg b.w. of glibenclamide also augmented the activity, which is similar to control animals exposed to vehicle 0.5% CMC (Table 5). Treatment of non-diabetic animals with 100 mg/kg b.w. naringenin did not change G6-PD activity compared to vehicle 0.5% CMC (Table 5).

#### Oral Administration of Naringenin Decreased the Activities of Glucose-6-Phosphatase and Fructose-1, 6-bisphosphatase in STZ-Injected Liver Tissues

Glucose-6-phosphatase (G6-P), a key gluconeogenic enzyme, catalyzes the dephosphorylation of glucose-6-phosphate to glucose (van Schaftingen and Gerin, 2002). Under normal conditions, insulin suppresses the activity of this enzyme (Shulman, 2000). However, in diabetics, the levels of G6-P get elevated to provide glucose to starving hepatocytes (Roden and Bernroider, 2003). Therefore, in order to test whether STZ induced animals also exhibit elevated G6-P activity, the method developed by Hikaru and Toshitsugu (1959) was adopted to measure the G6-P activity in the liver tissue (Koide, 1959). The inorganic phosphorus liberated during this conversion was estimated using Fiske and Subbarow (1925). The G6-P activity was significantly (*p* < 0.05) elevated in liver tissue of STZ control mice from 0.178 ± 0.02 units/mg protein to 0.297 ± 0.01 units/mg protein (Table 5). Elevated G6-P activity was reduced to about 0.255 ± 0.05 units/mg protein and 0.231 ± 0.01 units/mg protein, respectively upon 50 mg/kg b.w. and 100 mg/kg b.w. naringenin administration (Table 5). Oral administration of 600 μg/kg b.w. of glibenclamide decreased the activity to 0.202 ± 0.02 units/mg protein (Table 5). No significant change in G6-P activity was observed between vehicle control and 100 mg/kg b.w. naringenin-treated non-diabetic mice (Table 5).

Fructose-1, 6-bisphosphatase (F1,6-BP) is another key gluconeogenic enzyme effected by diabetes (van Schaftingen and Gerin, 2002). This enzyme is a rate-limiting enzyme in the gluconeogenic pathway and catalyzes the dephosphorylation of fructose-1,6-bisphosphate to fructose-6-phosphate (van Schaftingen and Gerin, 2002). The F1,6-BP activity in the liver tissue was determined by the method of Gancedo (Gancedo and Gancedo, 1971). The inorganic phosphorus liberated was estimated using (Fiske and Subbarow, 1925). Table 5 shows a significant increase in the activity of F1,6-BP from 0.189 ± 0.02 units/mg protein to 0.440 ± 0.03 units/mg protein upon STZ administration. Oral administration of 50 mg/kg b.w. naringenin and 100 mg/kg b.w. naringenin reduced the STZ induced F1,6-BP activity to 0.316 ± 0.01 units/mg protein and 0.267 ± 0.02 units/mg protein, respectively (Table 5). The positive control 600 μg/kg b.w. glibenclamide brought down the fructose-1, 6-bisphosphatase activity to 0.223 ± 0.02 units/mg protein (Table 5). However, treatment of control non-diabetic animals exposed to vehicle 0.5% CMC or treated with 100 mg/kg body weight naringenin did not change F1,6-BP activity (Table 5).

#### Oral Administration of Naringenin Increased the Liver Glycogen Levels in STZ-Administered Mice

Glycogen, a polymer of glucose residues produced by the glycogen synthase enzyme, is the key storage form of glucose and its amount in liver and muscle is an indication of insulin activity (Pederson et al., 2005). A method devolved by Morales et al. (1973) was used to extract and estimate the liver glycogen. Glucose is dehydrated by sulphuric acid to furfural derivative which then complexes with anthrone to give a green colored complex, which is read at 620 nm. The glycogen level was significantly (*p* < 0.05) decreased in liver tissues of STZ control mice from 32.44 ± 2.0 units to 21.27 ± 1.4 units (Table 5). Oral administration of 50 mg/kg b.w. naringenin and 100 mg/kg b.w. naringenin significantly augmented the glycogen content to 27.88 ± 1.6 units and 29.88 ± 0.9 units, respectively (Table 5). 600 μg/kg b.w. of glibenclamide also augmented to 28.05 ± 0.3 units. However, 100 mg/kg b.w naringenin-treated animals did not change liver glycogen compared to vehicle control treated non-diabetic mice (Table 5).

### Oral Administration of Naringenin Restored the Altered Morphology of Pancreatic Tissues Observed in Mice Administered With STZ

**FIGURE 8:**
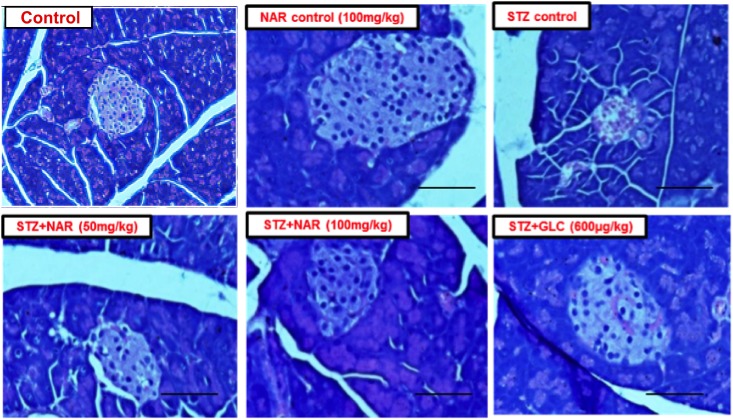
Administration of naringenin restored the normal structure of pancreas (Magnification: 40X, scale 100 μm). Control and naringenin treated animals exhibited normal pancreatic islet cells. However, animals administered with STZ showed a reduction in the size of islets, damaged β-cell population, and necrotic tissue. Analysis of pancreas collected from STZ administered mice subsequently treated with naringenin (50 mg/kg b.w.) exhibited moderate expansion of pancreatic islets, while other animals treated with 100 mg/kg b.w. naringenin restored necrotic and fibrotic changes and increased number and size of the islets. STZ mice treated with glibenclamide (600 μg/kg b.w.) showed absence of necrosis and fibrotic changes and increased number and size of the islets, indicating the restoration of normal morphological features.

Since STZ administration is known to damage pancreas by reducing the size and number of functionally active β-cells, an attempt was made to check whether naringenin and the positive control glibenclamide restored these altered morphological features (Bâlici et al., 2015). Control and naringenin (100 mg/kg b.w.) treated non-diabetic mice showed a normal pattern with clear and well-structured pancreatic islets (Figure 8). However, the pancreatic sections of STZ control mice displayed severe necrotic changes, exclusively in the center of pancreatic islets. In addition, disappearing of the nucleus with a comparative reduction in size was observed (Figure 8). Furthermore, pancreatic islets of STZ animals treated with naringenin (50 mg/kg b.w.) exhibited moderate expansion (Figure 8). Likewise, STZ animals treated with naringenin (100 mg/kg b.w.) showed a significant improvement in the pancreatic islet with distinct cellularity changes and increase in granulation, compared to the STZ control mice (Figure 8). The positive control 600 μg/kg b.w. glibenclamide showed healthy pancreas (Figure 8).

### Oral Administration of Naringenin Restored the Insulin Secreting Cells in Mice Administered With STZ

**FIGURE 9:**
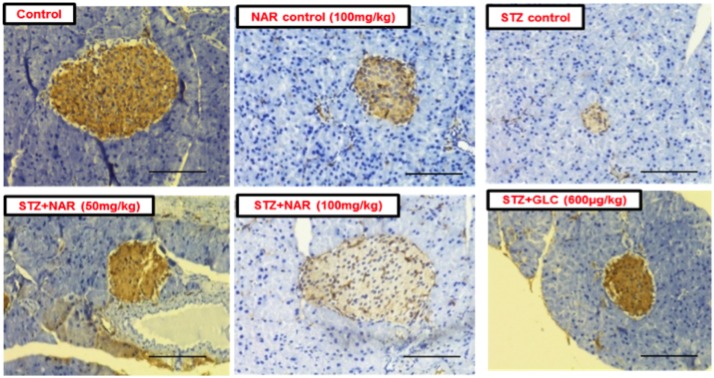
Immunohistochemical analysis of insulin secreting cells in pancreatic islets of normal and experimental mice (Magnification-400X, Scale-100 μm). Sections of pancreas collected from control and naringenin-treated non-diabetic animals showed the presence of normal morphology of islets and β-cells; however, STZ control showed a significant decrease in insulin immunoreactivity and a number of immunoreactive β-cells. While diabetic mice (STZ mice) treated with naringenin 50 mg/kg b.w. showed a moderate increase in insulin immunoreactivity, the ones administered with 100 mg/kg b.w. naringenin and 600 μg/kg b.w. glibenclamide showed a significant increase in insulin immunoreactivity and number of immunoreactive β-cells.

The immunohistochemical method was used to study the distribution pattern as well as number of functional β-cells in pancreatic islets of mice (Kim et al., 2009). Control (vehicle treated) and naringenin control (100 mg/kg b.w.) groups’ showed islets with a comparatively larger region of positive immuno-reactivity, demonstrating the existence of healthy cells secreting insulin in the pancreas (Figure 9). However, the STZ control islet cells showed distorted morphology with very few insulin-positive cells compared to vehicle controls (Figure 9). While 50 mg/kg b.w. naringenin showed a moderate increase in insulin immuno-reactivity, the ones received 100 mg/kg b.w. naringenin showed the relatively large area of positive immuno-reactivity with numerous brown insulin granules in the β-cells of pancreatic islets (Figure 9). Pancreatic section of STZ mice treated with positive control compound glibenclamide showed normal histo-architecture of the pancreas with insulin cells (Figure 9). Collectively, these observations conform the role of naringenin in increasing the insulin-positive cells in the pancreas.

## Discussion

Oxidative stress, which results from the imbalance due to: (a) overproduction of oxygen/nitrogen radicals; (b) loss/decrease of antioxidant enzymes; (c) low levels of natural endogenous antioxidants; (d) changes in transcription factors/transcription factor-associated protein(s) controlling the expression of endogenous radical scavenging enzymes (Rahal et al., 2014) results in the generation of oxidized lipids, broken DNA, modified proteins and carbohydrates (Birben et al., 2012). As a result of these changes in macromolecules: (a) the normal cells undergo transformation into cancer cells; (b) cells might lose normal functions such as on-demand proliferation and differentiation; (c) cells may undergo apoptosis or autophagic death, and (d) cells may lose the ability to execute repair processes (Klaunig et al., 2011). Therefore, oxidative stress is responsible for the origin of various diseases that include diabetes (Jiménez-Osorio et al., 2015). Hence, controlling oxidative stress is key to prevent diabetes.

Several lines of evidences suggest that phytochemicals such as flavonoids not only act as antioxidants to inhibit ROS but also trigger the expression of cyto-protective transcription factors such as Nrf2, which helps in the transcription of genes that include NQO1, SOD, GST, HO1 and many more (Stefanson and Bakovic, 2014). Among various flavonoids known to activate Nrf2 signaling resveratrol and curcumins have been well studied for treating diabetes (Tabatabaei-Malazy et al., 2015). Despite its potent role in Nrf2 activation, resveratrol suffers from low bioavailability, which limited its usage in the clinic (Elango et al., 2016). Hence, in this study, we made an attempt to investigate the Nrf2 activation potential of naringenin in STZ-exposed MIN6 cells and mice.

Pancreatic β-cells are highly susceptible to oxidative stress, which appears to be in part due to the lack of robust antioxidant capacity (Lenzen et al., 1996). Experimental studies have shown that adenoviral-mediated overexpression of antioxidant enzymes *in vitro* in β-cells, as well as exogenous treatment with antioxidants *in vivo* in animals, safeguards pancreatic β-cells from such insults (Lei and Vatamaniuk, 2011). Hence, initially, we checked whether naringenin has the potential to activate Nrf2 in MIN6 cell line using the Nrf2-Keap1 complementation system. The sensor system recognizes the potential of naringenin to promote Nrf2-Keap1 complex dissociation (Ramkumar et al., 2013). Treatment of MIN6 cell line with naringenin-induced endogenous as well as ectopically expressed Nrf2 in a dose-dependent manner indicating the potential of this compound to reduce oxidative stress. In addition, our data showed naringenin-induced activation and subsequent nuclear translocation of Nrf2 at a dosage of 50 and 100 μM. Further, when the MIN6 cells were transfected with luciferase-expressing ARE-NQO1 and ARE-GST constructs and, subsequently treated with naringenin, a dose-dependent increase in the luciferase activity in both NQO1 and GST was observed indicating that the naringenin treatment induced Nrf2, thereby elevated the levels of NQO1 and GST. Hence we further evaluated the antioxidant potential of naringenin in STZ induced diabetic models.

Streptozotocin, a derivative of *N*-methyl-*N*-nitrosourea, stands unique for its diabetogenic potential in β-cells (Schnedl et al., 1994). Mechanistically STZ induces diabetes by transferring the methyl group to the DNA molecule thereby triggering damage and fragmentation (Bennett and Pegg, 1981). DNA strand breaks induced by STZ treatment direct the cells to synthesize more poly (ADP-ribose) polymerase (PARP) to circumvent the STZ-induced apoptotic effects (Bennett and Pegg, 1981). However, over-stimulated PARP causes the reduction of intracellular NAD^+^ and ATP, which ultimately leads to β-cells apoptosis/necrosis (Szkudelski, 2001). Alternatively, it is also been proposed that STZ act as an intracellular nitric oxide donor, which stimulate the generation of ROS (Szkudelski, 2001). In the current study, MIN6 cells were exposed to a 10 mM concentration of STZ for 1 h to induce apoptosis (Kang et al., 2011). Since caspases-3 is involved in the PARP fragmentation, we have assessed the caspases-3 expression using western blotting. STZ induced the expression of caspases-3, however, naringenin attenuated the STZ induced apoptosis by suppressing the expression of cleaved caspases-3. Sustained production of ROSs is a primary stimulant of apoptosis. In the STZ induced MIN6 cells, we found elevated intracellular ROSs. Naringenin could decrease ROSs to a normal level by promoting the expression of NQO1 and GST via Nrf2 pathway.

*In vivo*, administration of naringenin significantly ameliorated the metabolic effects in STZ-induced diabetic mice. The raised blood glucose levels in diabetic mice were returned to near normal levels in diabetic mice treated with naringenin (Figure 6A). Likewise, the naringenin administration significantly enhanced the serum insulin level by stimulating the remaining pancreatic β-cells to produce more insulin to regulate the glucose level (Figure 6C). Further, naringenin showed dose-dependent improvement in the intraperitoneal glucose tolerance of the diabetic mice, which is comparable with the anti-diabetic drug, glibenclamide, a known stimulator of insulin (Figure 6B). Moreover, STZ-induced diabetic mice displayed significant abnormalities in lipid metabolism, which leads to significant elevation of serum cholesterol, triglycerides, LDL and VLDL and reduction in HDL levels. Administration of naringenin for 45 days showed a significant reduction in the serum cholesterol, triglycerides, LDL and VLDL and increased HDL levels in diabetic mice (Table 2). These results specify the lipid-lowering effect of naringenin on diabetic mice also the efficacy is comparative to the dose of naringenin.

Under physiological conditions, liver maintains normoglycemia by controlling blood glucose through glycolysis, gluconeogenesis, and glycogen synthesis processes (Han et al., 2016). However, due to insulin deficiency in diabetes, the above-stated functions are impaired and liver produces further glucose (Ozougwu et al., 2013). Few *in vivo* studies have shown that glucose homeostasis can be restored by phenolic compounds through changes in the activities of enzymes of carbohydrate metabolism in the liver of diabetic rats (Hanhineva et al., 2010; Chandramohan et al., 2015). In agreement with their study, in the current study, naringenin decreased the activities of key enzymes of gluconeogenesis such as glucose 6-phosphatase and fructose 1, 6-bisphosphatase in diabetic mice, increased the activity of glycolytic enzyme hexokinases, the activity of glucose 6-phosphate dehydrogenase and liver glycogen content. These modifications point the shifting of the metabolic pathways to reduced glucose production by the liver (Chandramohan et al., 2015). In addition, the effects of naringenin in the liver are accompanied by an increase in insulin expression as demonstrated by IHC, highlighting that insulin is responsible for these modifications. Furthermore, few recent studies have also shown that Nrf2 can reprogram the cells in such a way that the cell promotes synthesis (anabolism) rather than degradation (catabolism). Since naringenin increases Nrf2, the cells might have reoriented the cellular metabolism to synthesize more glycogen from glucose, leading to a significant decrease in blood glucose levels (Elango et al., 2016). In conclusion, results of our study clearly demonstrate that naringenin helps in the maintenance of glucose homeostasis by regulating key enzymes involved in the glucose metabolism, hence, naringenin may be considered as a good candidate drug for diabetes management.

## Conclusion

The *in vitro* and *in vivo* results of this study highlight the potential of naringenin to activate Nrf2 and protect the pancreatic β-cells from the oxidative damage caused by STZ. Above findings provide key evidences to demonstrate the anti-diabetic potential of naringenin. However, further studies testing the safety and efficacy of naringenin in higher animals are required to bring this natural product to the clinic. Additionally, strategies for improving the delivery of naringenin are also warranted to reduce the dose as well as to further enhance the potency.

## Author Contributions

RR conducted most of the experiments, analyzed the data, and wrote the manuscript. DS and MS performed and analyzed the experiments. SM, KR, and SS contributed to the experimental design and data and statistical analysis. All authors contributed to reviewing the results, writing the manuscript, and approved the final version of the manuscript.

## Conflict of Interest Statement

The authors declare that the research was conducted in the absence of any commercial or financial relationships that could be construed as a potential conflict of interest.

## Abbreviations

BSA: Bovine Serum Albumin
CAT: catalase
GPX: glutathione peroxidase
GSH: glutathione
IDF: International Diabetes Federation
IPGTT: Intraperitoneal Glucose Tolerance Test
Keap1: Kelch-like ECH-Associated Protein-1
MAPK: Mitogen-Activated Protein Kinase
MLD-STZ: Mutiple Low Dose Streptozotocin
NF-Kb: nuclear Factor kappa-light-chain-enhancer of activated B cells
NQO1: NAD(P)H Quinone Oxidoreductase 1
Nrf2: Nuclear factor E2-related factor 2
ROS: Reactive Oxygen Species
SDS-PAGE: Sodium Dodecyl Sulfate- Polyacrylamide gel electrophoresis
SOD: superoxide dismutase.
